# Impact of beef carcass size on chilling rate, pH decline, display color, and tenderness of top round subprimals

**DOI:** 10.1093/tas/txaa199

**Published:** 2020-10-30

**Authors:** Jessica M Lancaster, Brianna J Buseman, Tanya M Weber, James A Nasados, Ron P Richard, Gordon K Murdoch, William J Price, Michael J Colle, Phillip D Bass

**Affiliations:** 1 Department of Animal and Veterinary Sciences, University of Idaho, Moscow, ID; 2 Statistical Programs, College of Agricultural and Life Sciences, University of Idaho, Moscow, ID

**Keywords:** beef, carcass size, color, pH, temperature, top round

## Abstract

Beef carcass weights in the United States have continued to increase over the past 30 yr. As reported by the United States Department of Agriculture, grid-based carcass weight discounts begin when carcasses exceed 408 kg. Despite weight discounts, beef carcass weights continue to increase. At the same time, an increased prevalence of discoloration and color variability in top round subprimals has been observed throughout the industry which may be influenced by the increases in carcass weights. The objectives of this study were to assess the effects of beef carcass size and its relationship to chill time, color, pH, and tenderness of the beef top round. In the current study, eight industry average weight beef carcasses (AW, 341–397 kg) and eight oversized beef carcasses (OW, exceeding 432 kg) were evaluated. Temperatures and pH measurements were observed on both sides of all carcasses for the initial 48 h postharvest at a consistent superficial and deep anatomical location of the respective top rounds. Carcasses were fabricated into subprimals at 48 h and top rounds were aged at 2 °C for an additional 12 d. The superficial location of both AW and OW carcasses cooled at a faster rate (*P* < 0.01) than the deep locations. The deep location of OW carcasses had a lower pH and a more rapid (*P* < 0.01) initial pH decline. Quantitative color of steaks from OW carcasses had greater mean L* (lightness; *P* = 0.01) and initial b* (yellowness; *P* < 0.01) values. The delayed temperature decline and the accelerated pH decline of the deep location of the top round of OW carcasses occur at different rates than AW carcasses. Delayed rate of cooling leads to irreversible impacts on steak appearance of top round steaks fabricated from OW beef carcasses when compared with AW carcasses.

## INTRODUCTION

The average beef carcass weight in the United States has increased over 61 kg in the past 30 yr (USDA-ERS, 2020). On a grid-based marketing system, carcass weight discounts are applied once carcass weights reach the threshold of 408 kg (USDA-AMS, 2020). The 2016 National Beef Quality Audit reported that 32.2% of beef carcasses observed had hot carcass weights exceeding 408 kg with a maximum weight of 615 kg (Boykin et al., 2017). Even with the carcass weight discounts, the beef industry in North America continues to see incremental increases in fed beef carcass weights as producers can achieve greater gross profits on heavy weight carcasses because of the additional weight produced. As the number of heavy weight carcasses continue to increase in the beef industry, the researchers postulated that additional focus should be placed on thicker areas like the round to avoid potentially negative effects on meat quality associated with cooling.

The semimembranosus (SM) is a muscle that starts at the highest temperature and takes longer to dissipate heat than other muscles on the carcass (Hannula and Puolanne, 2004). Slower temperature declines postmortem result in accelerated pH declines, which suggests temperature and pH are not independent parameters (Mohrhauser et al., 2014). Furthermore, delayed temperature declines in combination with elevated pH results in meat that is paler in color with decreased protein functionality resulting in decreased water holding capacity (Jacob and Hopkins, 2014). Discoloration has been observed in the SM at the location closest to the femur bone and can be attributed to myoglobin denaturation due to high temperature, low pH conditions, and resulting in enzyme denaturation leading to potential tenderness issues as well (Kim et al., 2010). The temperature gradient and pH decline between the deep and superficial locations can result in negative impacts to color and meat quality attributes. Thus, the objective of the current study was to assess the effects of beef carcass size and its relationship to chill time, color, pH, and tenderness of the beef top round. The researchers hypothesized that the top rounds from OW carcasses would cool at a slower rate with a more rapid pH decline and subsequently yield top round steaks that were less color stable and less tender than AW carcasses.

## MATERIALS AND METHODS

### Product Procurement

Between September 2018 and April 2019, beef steer carcasses that were within the parameters of United States Department of Agriculture (USDA) Yield Grade 2 or 3 were selected for the study. Steers were harvested at the University of Idaho Meat Laboratory under USDA-Food Safety Inspection Service inspection humane animal harvesting guidelines as outlined by Title 9 of the Code of Federal Regulations part 313. Carcasses were identified in the harvesting facility by the hot carcass weight (HCW) as “average weight” (AW) (*n* = 8, 335–387 kg), and “oversized” (OW) (*n* = 8, ≥ 432 kg). Carcasses considered AW in this study would not receive a discount for carcass weight on a grid-based value pricing system, whereas OW carcasses receive a discount because of HCW according to USDA carcass price reporting (USDA-AMS, 2020). All carcasses were from typical commercially raised concentrate-finished *Bos taurus* beef cattle; due to the commercial nature of production and purchasing, cattle rations were not available.

### Temperature and pH Decline

Following USDA final inspection (approximately 35 min postmortem), a temperature monitoring data-logging probe (Multi-trip, Tempcord, Tulsa, OK) was inserted into the semimembranosus (SM) at the approximate geometric center of the round medially until making contact with the femur bone of the respective carcass. This anatomical location was considered the “deep” location. An additional temperature monitoring data-logging probe was inserted 2.54 cm from the surface of the top round in accordance with industry standard for temperature monitoring for Hazard Analysis and Critical Control Point plans (USDA-FSIS, 2017). This anatomical location was considered the “superficial” location. Temperature monitoring data logging probes were placed in both sides of the carcass and positioned as described. Temperature decline was logged every 30 s and recorded over a 48-h period during the chilling process. Temperature and pH values were averaged from the two sides of each carcass. The pH of deep and superficial locations of the top round was measured with a portable pH meter (Apera Instraments SX811-SS, Columbus, OH) with a puncture-type probe every h for the first 12 h and then every 6 h until 48 h following initial entry into the cooler. Superficial and deep locations of the top round were measured on both sides of each carcass at all timepoints. Calibration standards (4.0, 7.0, 10.0) (Hanna Instruments, Woonsocket, RI) were utilized prior to measurements. Sides were evenly spaced in a 6.1 m × 2.1 m chill cooler (0 °C) and placed to avoid touching other sides or infrastructure. Carcasses were ribbed between the 12th and 13th rib at 48 h postmortem. The pH of the ribeye at the 12th to 13th rib interface was assessed to ensure that it was within the normal range of 5.4 to 5.7 (Matarneh et al., 2017). Quality grade was determined on each side by trained University of Idaho personnel using USDA Quality Grade standards. Dentition was used to classify all carcasses as under 30 mo of age. Ribeye area and external fat thickness (adjusted) were measured (external fat thickness, 3/4 of the length of the Longissimus dorsi muscle from the chine bone) on each side. Kidney, pelvic, and heart fat percentages were estimated using visual estimation.

### Preparation of Product

After 48 h chilling postmortem at 0 °C, carcasses were fabricated to obtain the top round subprimal (Institutional Meat Purchase Specification #168) from both sides of each carcass. Maximum circumference of the round (aitch bone removed) was recorded using a flexible tape measure. Top rounds were weighed, denuded, and the cap and side muscles were removed (NAMA #169A; NAMA, 2014). The trimmed top rounds were weighed, measured (length and width), vacuum packaged, and aged for an additional 12 d at 2 °C. After the subprimal aging period, six steaks were cut to 2.54 cm thicknesses and were fabricated proximal to distal, perpendicular to the longitudinal axis of the cut. Ultimate pH was recorded at a point 2.54 cm from the deep and superficial side of each steak. All steaks were individually weighed. Four steaks from each subprimal were randomly and evenly assigned to a shelf-life treatment (d 1, d 2, d 3, or d 4 of shelf life). Each steak was placed in a commercially available rose-colored foam meat tray (23.8 cm × 31.4 cm; Bunzl; Riverside, MO) and overwrapped using an oxygen permeable polyvinyl chloride film (oxygen transmission: 1450 cc/645 cm^2^ per 24 h, water vapor transmission rate: 17.0 g/645 cm^2^ per 24 h, Koch Industries, Inc. #7500-3815; Wichita, KS). During steak fabrication, one steak was vacuum packaged and frozen at −20 °C for later quantitative Warner–Bratzler shear force (WBSF) tenderness analysis. A 10 g sample was removed from deep and superficial locations of the top round on d 2 and d 14 postmortem for calpain activity analysis. Samples from differing time periods were acquired independently. These samples were snap frozen in liquid nitrogen, stored in conical tubes (15 mL, VWR Centrifuge Tubes, Radnor, PA), and frozen at −76 °C until measurement for calpain activity. Immediately following exsanguination, a single sample of the Sternocephalicus muscle was obtained from a reference steer carcass and snap frozen to be utilized as a reference sample for calpain analysis.

### Retail Color

Before objective color measurements, steaks were packaged and allowed to bloom for 60 min. Two objective color measurements per location (deep and superficial) were recorded using a portable hand-held spectrophotometer (MiniScan EZ, HunterLab, Restin, VA). Subsequent color measurements were taken on d 1, d 2, d 3, and d 4. Caution was taken during sampling to avoid large marbling flecks and connective tissue. The spectrophotometer was equipped with a 25 mm-diameter opening and a 10° standard observer. The instrument was set to illuminant A and Commission International de I’Eclairage (CIE) L* (lightness), a* (redness), and b* (yellowness) values were recorded. Calibration of the spectrophotometer was performed daily utilizing calibration tiles (white and black) through the packaging film prior to analyzing color on the meat samples.

In addition to objective color measurements, steaks were visually evaluated on d 0, d 1, d 2, d 3, and d 4 of retail display. Parameters measured via visual evaluation of subjective color were oxygenated lean color, amount of browning, discoloration, surface discoloration, and color uniformity by at least three trained color evaluators following American Meat Science Association (AMSA) Meat Color Measurement Guidelines (AMSA, 2012). Steaks were displayed in a glass-fronted, sliding door, retail display case (Model GDM-69, True Manufacturing Co., O’Fallon, MO) at 3 °C for the duration of the retail display. The display case was lit with natural white Hg 40 W fluorescent lights which were on for the duration of the retail display with an average light intensity of 401 lx (Fisherbrand Traceable Dual-Range Light Meter, Fisher Scientific, Waltham, MA). To avoid potential confounding effects due to display case locations, steak positions were rotated after each measurement. Steaks from the front of the case were moved to the back and raised to the next shelf up while steaks on the top shelf would rotate to the bottom; steaks in sequential locations followed suit until the retail display period was finished.

### Lipid Oxidation

Thiobarbituric acid reactive substances (TBARS) were analyzed on d 0, d 1, d 2, d 3, and d 4 of retail display following the protocol provided in Appendix O of the Meat Color Measurement Guidelines (American Meat Science Association, 2012). The samples (deep and superficial locations) were taken from the exposed surface of the steak, 1.27 cm deep into the steak, and avoided the edge of the steak following the procedure as previously described by Colle et al. (2016). Briefly, steaks assigned to the respective day were sampled by removing the edge of the steak (approximately 1 cm) and samples measuring 0.5 cm wide × 2.0 cm long × 1.27 cm thick were utilized for the analysis.

### Warner–Bratzler Shear Force

Warner–Bratzler shear force (WBSF) steaks were tempered for 24 h at 4 °C before cooking on a two-sided, clamshell style electric grill (Cuisinart Griddler Deluxe Model GR-150) to a target internal temperature of 71 °C. Peak internal temperature was recorded using a Type K thermocouple (Copper-Atkins 93230-K EconoTemp). Times were recorded for when steaks were placed on grills, removed from grills, and when steaks reached peak temperatures. Five cores (1.27 cm diameter) were removed parallel to the muscle fiber orientation for the deep and superficial locations of each steak. Each core was sheared once perpendicular to the muscle fiber orientation using a WBSF machine (G-R Manufacturing, Manhattan, KS). Peak shear force values were used to compute a mean peak shear force value of each steak. Steaks were weighed before and after cooking to determine percentage cook loss.

### Calpain Extraction and Casein Zymography

Extraction buffer (3 mL, 100 mM Tris, 10 mM EDTA, 10 mM DTT, pH 8.3) was added to one gram of frozen sample previously stored at −75 °C and homogenized (Polytron^®^ PT 10–35 GT; PT-DA 12/2EC-B154) at 18,000 rpm on ice three times for 15 s with cooling (15 s) between bursts. The homogenate was pipetted into 2 mL microcentrifuge tubes. Subsequently, samples were centrifuged for 30 min at 8,800 × *g* at 4 °C. The supernatant fluid was aliquoted and stored until calpain analysis at −75 °C.

 Calpain-1 and calpain-2 activity was determined utilizing casein zymography as originally described by Raser et al. (1995) and expanded upon by Pomponio et al. (2008) and Pomponio and Ertbjerg (2012) with minor modifications. Polyacrylamide gels (12.5%; 75:1 acrylamide to bisacrylamide) containing 0.2% casein were poured and overlaid with stacking gel (4%; 75:1 acrylamide to bisacrylamide) on the day the gels were run. Gels (8 × 10 × 0.1 cm) were pre-run with running buffer (25 mM Tris, 1 mM DTT, 192 mM glycine, 1 mM EDTA, pH 8.3) at 100 V for 15 min in an ice bath before loading samples. Sample buffer (10 µL) (150 mM Tris, 20% glycerol, 10 mM DTT, 0.02% bromophenol blue, pH 6.8) was added to the supernatant (40 µL). Samples (20 µL) were added and the gels were run at 100 V for 6 h in an ice water bath. Gels were then placed in incubation buffer (~60 mL; 50 mM Tris, 10 mM DTT, 4 mM calcium chloride, pH 7.5) at room temperature with slow shaking for 17 h. Buffer was changed (~60 mL) at 30 min and (~130 mL) 60 min. Gels were stained in a commercially available, premixed, Coomassie Blue R-250 for 1 h and destained in a commercially available, premiex Coomassie Blue R-250 destaining solution for 3 h. The clear bands indicating calpain activity were quantified by inverting the image and then comparing the density of each band to the d 0 sample on each gel utilizing a ChemiDoc MP™ System (BioRad). Autolysis was used as an indicator of calpain activation (Geesink et al., 2006). Calpain activity was evaluated individually on for the deep and superficial portion of the top round from each side of each carcass at both d 2 and d 14. Calpain band densities were expressed as a percentage of the d 0 sample (collected immediately post exsanguination) of Sternocephalicus as an indicator of calpain activation.

### Statistical Analysis

Temperature and pH data were modeled using nonlinear regression following the exponential form: 

$$\begin{array}{l} y_{i}=a*exp(-b*hour_{i})+c, \end{array}$$

where *y*_*i*_ is the temperature or pH response at the *i*th hour, *c* is a lower asymptote, *a* + *c* represents the intercept term, and *b* is the rate of change over time (hour). This model describes a general decline in response over time starting at a value of *a* + *c* which eventually flattens out at a lower value *c*. Larger values of the rate parameter indicate a faster decline. Each treatment was initially estimated separately to ensure an adequate fit to eq (1). Subsequently, a full model incorporating all treatments was fit allowing pair-wise comparison of model parameters across treatments.

All other data were analyzed using Mixed Model procedure of the Statistical Analysis System (SAS) assuming a randomized complete block design with treatments as fixed effects. Differences in the least squares means (LSM) were compared using pair-wise comparisons. Statistically significant p-values were evaluated at *P* < 0.05. Carcass served as the experimental unit with side as a replicate. Repeated measures were used for color analysis with day as the repeated measure and steak as the subject. Calpain activity is presented as a relative value and not in absolute terms. All data were analyzed using SAS version 9.4 (SAS Institute, Inc., Cary, NC).

## RESULTS

**Figure 1.**
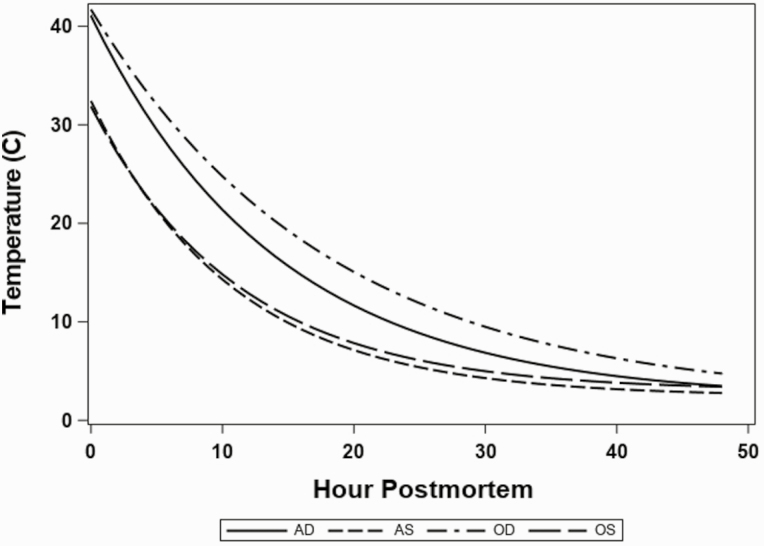
Estimated models of temperature (°C) of top round subprimals (semimembranosus) decline over 48 h chill (starting immediately after final inspection, ~35 min postmortem) period for average and oversized carcasses monitoring the deep and superficial location. Individual lines [average superficial (AS), average deep (AD), oversized superficial (OS), oversized deep (OD)] estimated following the model: temperature = a*exp(−b*hour) + *c*.

Temperature decline data for each treatment fit well to their respective models (Supplementary Figure 1) and all parameter estimates were significant (*P* < 0.01). The overall difference between the initial top round temperatures, *a* + *c*, for AW and OW (36.72 and 36.77 °C, respectively) was not different (*P* = 0.70), however the AW cooled at a faster rates (*P* < 0.01) than OW (Figure 1). The deep versus superficial starting temperatures (41.73 and 32.13 °C, respectively) were different (*P* < 0.01) and the rate of change over time were also different (*P* < 0. 01) with the superficial locations having a greater rate of change over time than the deep locations (Figure 1). Furthermore, there was a difference (*P* < 0.01) in the temperature rate of change between the OW deep location that cooled at a slower rate than the AW deep location (Figure 1). There was not a difference (*P* = 0.38) between the rate of temperature change of the OW superficial and AW superficial locations (Figure 1). Temperature at 48 h was not different (*P* = 0.55).

**Figure 2.**
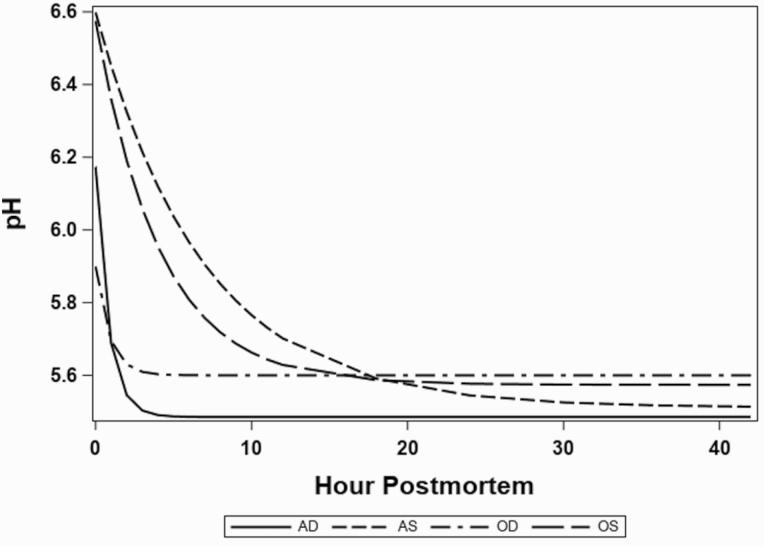
Estimated models of pH decline of top round subprimals decline over 48 h chill (starting immediately after final inspection, ~35 min postmortem) period for average and oversized carcasses monitoring the deep and superficial location. Individual lines [average superficial (AS), average deep (AD), oversized superficial (OS), oversized deep (OD)] estimated following the model: pH = a*exp(−b*hour) + *c*.

Th pH decline data for each treatment fit well to their respective parameters (Supplementary Figure 2). The overall starting pH, *a* + *c*, of the top round for AW and OW (6.39 and 6.24, respectively) carcasses was different (*P* < 0.01) and the starting pH for deep versus superficial locations (6.04 vs. 6.59) was different (*P* < 0.01; Figure 2). The pH rate of decline was different (*P* = 0.02) with the deep locations declining much more rapidly than the superficial locations (Figure 2). Furthermore, there was a difference (*P <* 0.01) in the rate of pH change between the OW deep location and the AW deep location, the OW deep location declining the fastest (Figure 2). However, ultimate pH (48 h) was not different (*P* = 0.26) between AW and OW carcasses (Figure 2).

Analysis of carcass traits are presented in Table 1. Hot carcass weights of the AW were lighter (*P* < 0.01) than the OW carcasses. 12th rib fat thickness and KPH fat percentage were not different between AW and OW carcasses (*P* = 0.24 and 0.79, respectively), but OW carcasses had larger ribeye areas (*P* < 0.01). In addition, calculated yield grade was not different (*P* = 0.47) between AW and OW carcasses. Intact round circumference of AW carcasses was smaller (*P* < 0.01) than the round circumference of OW carcasses. Top round length and width was greater (*P* < 0.01) for OW carcasses than AW carcasses. Untrimmed top round weight was greater (*P* < 0.01) for OW carcasses than AW carcasses. Trimmed top round weight tended to be greater for OW than AW carcasses however, it was not different (*P* = 0.07; Table 1).

**Table 1.** Carcass trait means of steers (*N* = 16) and top round subprimal characteristics (*n* = 32)

	Average			Oversized			
Carcass	Min	Max	Mean	Min	Max	Mean	SEM
Hot carcass weight, kg	335	387	366^b^	432	500	462^a^	6
12th rib fat thickness, cm	0.76	2.03	1.32	1.02	2.29	1.50	0.12
Ribeye area, cm^2^	68.4	103.2	85.9^b^	83.9	107.1	98.4^a^	2.5
Kidney, pelvic, and heart fat, %	1.5	2.5	2	1.5	2.5	2	0.1
Round circumference, cm	109.2	128.3	117.3^b^	121.9	134.6	128.5^a^	1.3
Calculated yield grade	2.46	3.98	3.24	2.94	3.92	3.35	0.11
Top round subprimal							
Length, cm	40.6	53.3	49.5^b^	58.4	76.7	55.6^a^	0.8
Width, cm	52.8	63.5	57.7^b^	58.4	73.7	65.3^a^	1.0
Untrimmed wt., kg	9.04	11.62	10.32^b^	10.94	14.75	13.07^a^	0.24
Trimmed wt., kg	5.57	8.82	6.85	6.37	9.02	7.51	0.23
Ultimate pH	5.4	5.6	5.5	5.4	5.6	5.5	0.02

^ab^Within a trait, means without common superscripts differ (*P* < 0.05).

Calpain-1 activity was observed in the reference muscle (Sternocephalicus, d 0 postexsanguination), however calpain-1 activity was not observed at all in the SM samples (data not shown). Calpain-2 activity was greater (*P* = 0.01) for superficial locations than the deep locations based on values relative to the zero-hour reference standard (89.44% and 68.24%, respectively; Table 2). Autolyzed calpain-2 activity was greater (*P* = 0.04) in OW carcasses compared to AW carcasses.

**Table 2.** Means of native calpain-2 and autolyzed calpain-2 activity of semimembranosus from average and oversized carcasses (*n* = 32) across all aging periods

	Average	Oversized	SEM
Native calpain-2 activity^*^	81.5	76.2	5.9
Autolyzed calpain-2 activity^*^	5.1^b^	14.0^a^	5.1
	Deep^†^	Superficial^†^	SEM
Native calpain-2 activity^*^	68.2^b^	89.4^a^	5.8
Autolyzed calpain-2 activity^*^	10.0	4.5	4.7

^*^As a percentage of day 0 calpain-2 activity.

^†^Averaged among average and oversized carcasses.

^ab^Within a trait, means without common superscripts differ (*P* < 0.05).

Analysis of lipid oxidation values indicated a two-way interaction (*P <* 0.01) between day and carcass weight (Table 3). Generally, all treatment combinations increased in lipid oxidation over days of retail display, with the AW carcasses having greater TBARS values starting at d 2.

**Table 3.** Mean values of lipid oxidation^*^ for top round steaks (*n* = 32) of average and oversized carcasses over a 4-d simulated retail display

	Day of retail evaluation					
	0	1	2	3	4	SEM
Average	0.17^c^	0.29^b^	0.36^b x^	0.56^a x^	0.64^a x^	0.04
Oversized	0.10	0.20	0.20^y^	0.23^y^	0.27^y^	0.04

^*^mg malondialdehyde/kg meat.

^abc^Within a carcass weight, means without common superscripts differ (*P* < 0.05).

^xy^Within a day, means without a common superscript differ (*P* < 0.05).

There was a weight by day interaction for objective color scores for L* (*P* = 0.01), a* (*P* < 0.01), and b* (*P* < 0.01) (Table 4). The OW carcasses were lighter in color compared to the AW carcasses on d 0, d 2, and d 4. Redness (a*) decreased throughout the retail display for both AW and OW carcasses. The deep location of steaks from OW carcasses was lightest in color compared to all other locations (Table 6). On d 1, steaks from OW carcasses were redder in color (a*; *P* < 0.01) whereas on d 2 they were less red compared to AW steaks. The superficial location on steaks remained more red (a*; *P* < 0.01) from d 1 to d 4 of the retail display when compared to the deep location (Table 5). There was a difference in b* values (*P* < 0.01) with the steaks from OW carcasses being more yellow in appearance on the initial 2 d compared to steaks from AW carcasses (Table 4). Subjective color scores increased (favorable color to unfavorable color) from d 0 to d 1 for: oxygenated lean color (*P* < 0.01), browning (*P* < 0.01), discoloration score (*P* < 0.01), degree of surface discoloration (*P* < 0.01), and steak uniformity (*P* < 0.01) (Table 7). Steaks from AW carcasses had greater oxygenated lean scores (*P* = 0.05) and less surface discoloration (*P* = 0.02) when compared to OW carcasses (Table 8).

**Table 4.** Objective color measurement means for L*, a*, and b* of top round steaks (*n* = 32) of average and oversized carcass over a 4-d simulated retail display

	Day of retail evaluation					
	0	1	2	3	4	SEM
L*						
Average	37.84^b w^	37.50^wx^	34.38^b y^	37.07^wxy^	36.16^b xy^	0.87
Oversized	40.99^a w^	39.15^x^	39.23^a x^	39.09^x^	39.64^a wx^	0.86
a*						
Average	25.67^w^	24.22^b x^	26.08^a w^	20.90^y^	20.60^y^	0.63
Oversized	27.27^w^	26.03^a w^	22.88^b w^	21.00^y^	19.43^z^	0.62
b*						
Average	22.54^b x^	21.91^b x^	26.22^a w^	21.76^x^	21.87^x^	0.50
Oversized	27.00^a w^	26.37^a w^	23.45^b x^	22.57^xy^	21.82^y^	0.50

^ab^Within carcass weight, means without common superscripts differ (*P* < 0.05).

^xy^Within a day within a trait, means without common superscripts differ (*P* < 0.05).

**Table 5.** Objective color measurement means for a* of the deep and superficial location of top round steaks (*n* = 32) averaged across average and oversized carcass over a 4-d simulated retail display

	Day of retail evaluation					
	0	1	2	3	4	SEM
Deep	25.93^a^	23.27^b y^	22.07^b y^	18.55^c y^	17.65^c y^	0.58
Superficial	27.01^a^	26.99^a x^	26.89^a x^	23.35^b x^	22.38^b x^	0.56

^abc^Within a steak location, means without common superscripts differ (*P* < 0.05).

^xy^Within a day, means without common superscripts differ (*P* < 0.05).

**Table 6.** Objective color measurement means for L* of top round steaks (*n* = 32) from average and oversized carcasses averaged across a 4-d simulated retail display period for the deep and superficial locations

	Average	Oversized	SEM
Deep	38.98^a y^	43.23^a x^	0.78
Superficial	34.20^b^	36.00^b^	0.77

^ab^Within a carcass weight, means without common superscripts differ (*P* < 0.05).

^xy^Within a steak location, means without common superscripts differ (*P* < 0.05).

**Table 7.** Subjective color score means of top round steaks (*n* = 32) from average and oversized carcasses across a 4-d simulated retail display period

	0	1	2	3	4	SEM
Oxygenated lean^*^	4.12^e^	4.30^d^	4.56^c^	5.32^b^	5.81^a^	0.14
Browning^†^	1.70^e^	2.47^d^	2.93^c^	3.43^b^	3.74^a^	0.12
Discoloration Score^||^	2.03^e^	2.67^d^	3.05^c^	3.53^b^	3.93^a^	0.11
Surface discoloration^$^	2.28^e^	2.95^d^	3.24^c^	3.75^b^	4.20^a^	0.13
Uniformity^¶^	2.58^e^	2.84^d^	3.20^c^	3.58^b^	3.87^a^	0.12

^*^1 = extremely bright cherry-red, 2 = bright cherry-red, 3 = moderately bright cherry-red, 4 = slightly bright cherry-red, 5 = slightly dark cherry-red, 6 = moderately dark red, 7 = dark red, 8 = extremely dark red.

^†^1 = no evidence of browning, 2 = dull, 3 = grayish, 4 = brownish-gray, 5 = brown, 6 = dark brown.

^||^1 = none, 2 = slight, 3 = small, 4 = moderate, 5 = extreme.

^$^1 = no discoloration (0%), 2 = slight discoloration (1% to 20%), 3 = small discoloration (21% to 40%), 4 = modest discoloration (41% to 61%), 5 = moderate discoloration (61% to 80%), 6 = extensive discoloration (81% to 100%), reported as average of steaks from average and oversized carcasses.

^¶^1 = uniform, no two-toning, 2 = slight two-toning, 3 = small two-toning, 4 = moderate two toning, 5 = extreme two-toning, reported as average of steaks from average and oversized carcasses.

^abcde^Within a trait, means without common superscripts differ (*P* < 0.05).

**Table 8.** Subjective color score means of top round steaks (*n* = 32) from average and oversized carcasses averaged across a 4-d simulated retail display period

	Average	Oversized	SEM
Oxygenated lean^*^	5.09^a^	4.70^b^	0.14
Surface discoloration^†^	3.04^b^	3.53^a^	0.14

^*^1 = extremely bright cherry-red, 2 = bright cherry-red, 3 = moderately bright cherry-red, 4 = slightly bright cherry-red, 5 = slightly dark cherry-red, 6 = moderately dark red, 7 = dark red, 8 = extremely dark red.

^†^1 = no discoloration (0%), 2 = slight discoloration (1% to 20%), 3 = small discoloration (21% to 40%), 4 = modest discoloration (41% to 61%), 5 = moderate discoloration (61% to 80%), 6 = extensive discoloration (81% to 100%).

^ab^Within a trait, means without common superscripts differ (*P* < 0.05).

Carcass weight did not impact cook time (*P* = 0.64), percent cook loss (*P* = 0.48), or WBSF (*P* = 0.16) values (Table 9). The location within the steak also did not impact WBSF (*P* = 0.14; Table 9).

**Table 9.** Mean percent cook loss, cook time, and WBSF of top round steaks (*n* = 32) from average and oversized carcasses

	Average	Oversized	SEM
Percent cook loss	31.32	29.50	1.79
Cook time*	7.22	7.40	0.26
WBSF, kg	3.83	3.62	0.11
	Deep^†^	Superficial^†^	
WBSF, kg	3.84	3.61	0.11

^*^Min to 71 °C, measured at the geometric center.

^†^Averaged among average and oversized carcasses.

## DISCUSSION

The top round from AW and OW carcasses started at similar temperatures, but AW carcasses cooled at a faster rate than OW carcasses. Similar results have been observed by others modeled in the Longissimus lumborum (Agbeniga and Webb, 2018; Fevold et al., 2019) and SM (Djimsa et al., 2018; Fevold et al., 2019; Klauer, 2019). The deep location had a greater temperature change from initial to final temperature than the superficial location, similar to data reported by Sammel et al. (2002b). In the present study, there was no difference in the magnitude of temperature change from initial to final temperature of deep locations within AW and OW carcasses (38.91 and 39.70 °C, respectively), however, the deep location of AW carcasses cooled at a faster rate than that of OW carcasses. Contrary to the current study, previous research reported no difference in the temperature decline of AW and OW carcasses at the center of the SM, measured 12.7 cm below the surface (Klauer, 2019). However, no difference was observed in the current research between the AW and OW superficial location temperature decline which is in agreement with previous research (Klauer, 2019). The superficial position of the SM is the location most often used for in-plant Hazard Analysis Critical Control Points temperature monitoring (USDA-FSIS, 2017); the data from the current study would indicate that it would still be an effective location for monitoring temperature decline regardless of carcass weight. Hot boning the top round has been suggested as an alternative to overcome challenges with chilling rate of the deep portion of the SM and the subsequent effects on pH decline (Sammel et al., 2002b), but this practice has not been widely implemented in the industry (Troy, 2006). Disadvantages associated with hot boning have included: infrastructure or changes to current plant processes, increased microbial load, decreased product tenderness, and subsequent impacts on eating experience (Troy, 2006).

The pH decline for the superficial location of the AW and OW carcasses was similar in the current study. However, the deep location for both carcass weights started at a lower initial pH with the OW carcasses starting lower than the AW carcasses (5.90 vs. 6.18, respectively). Agbeniga and Webb (2018) reported similar trends in the decline of the Longissimus lumborum muscle pH where OW carcass pH decreased faster than that of AW carcasses. Djimsa et al. (2018) reported OW carcasses had a more rapid pH decline in the Longissimus dorsi, Psoas major, and SM compared with AW carcasses. Meanwhile, Fevold et al. (2019) reported no difference in pH decline among carcasses of different weights at h 0, h 4, and h 24 measured at the center of the SM. In addition, results from the current study indicate the deep location of the inside round started at a lower initial pH than the superficial location. Sammel et al. (2002b) also reported the deep location (10 cm below surface of muscle) pH declined more rapidly in the initial hours than the superficial location. Furthermore, in agreement with Sammel et al. (2002b), intermediate pH declines changing at different rates did not impact the ultimate pH and fell within a normal range of around 5.6 for ultimate muscle pH. Similar patterns have been reported in the biceps femoris; the inner location of the muscle dropped at a faster rate than the more exterior location (Kuffi et al, 2018). Temperature plays an important role in the decline of pH; higher temperature conditions result in a faster rate of energy substrate utilization, hence a greater accumulation of hydrogen ions reflected as a lowered pH (Matarneh et al., 2017). Previously, den Hertog-Meischke et al. (1997) reported that when pH values had reached below 6.0 while the temperature remained above 30 °C, it resulted in greater denaturation of myosin. For both the AW and OW carcass deep locations in the current study, a similar pH and temperature relationship was observed, with the pH being even more dramatically decreased in the OW carcasses. This decrease in pH could lead to further denaturation implications and negatively impact color, water holding capacity (Jacob and Hopkins, 2014) and enzymatic tenderness (Hwang and Thompson, 2001).

Despite differences in carcass weight, the back fat, KPH, and calculated yield grade were not different between the AW and OW carcass treatments. These results are different from previous research in that additional carcass weight was associated with increased back fat and KPH (Klauer, 2019). The current study was designed to target carcasses with similar yield grades in an effort to account for 12th rib fat thickness and muscling amount while previous work by Klauer (2019) and Fevold et al. (2019) did not select carcasses on these parameters but rather on HCW alone. On average, carcasses from the OW treatment had larger ribeyes compared with AW carcasses, as seen in previous research (Fevold et al., 2019; Klauer, 2019). Top round subprimals and subsequent trimmed, steak-ready subprimal weights were greater for OW carcasses than AW carcasses. Although top round subprimals were significantly larger for the OW carcasses, once the cap was removed and the SM was denuded, the differences between the respective muscles were no longer significant. It is possible that the increased size of the cap muscles caused a greater fat coverage on the medial section of the top round of the larger weight carcasses. Nevertheless, those measurements were not recorded in the present study.

Slower chilling rates of the Longissimus lumborum in previous studies have resulted in a decrease of extractable calpain-1 and calpastatin at 24-h postmortem as a result of rapid glycolysis (Hwang and Thompson, 2001). In the current study, calpain-1 activity was not observed at all in the SM samples likely due to the collection of those samples occurring at 48-h postmortem. Native calpain-2 across all time points (2 and 14 d) was observed in greater activity in superficial locations compared with the deep locations. Colle and Doumit (2017) reported the relative percentage of native calpain-2 from the proximal location of the SM aged for 14 d was less than the superficial and deep locations examined in this study. In addition, a greater activity of autolyzed calpain-2 in OW carcasses were observed in the current study. The autolyzed calpain-2 activity in the AW carcasses of the current study was similar to that reported by Colle and Doumit (2017) which were obtained from carcasses that were not selected for specific weight criteria. The OW carcasses had almost triple the relative percentage of autolyzed calpain-2 activity previously observed at 14 d from commercially sourced product (Colle and Doumit, 2017). The relative percentage of autolyzed calpain-2 activity in the OW carcasses of the current study was more similar to observed levels in extended aged (42 d) product from the SM sourced from more traditional carcass sizes (Colle and Doumit, 2017). The elevated temperature for a greater length of time during the initial chilling process could have contributed to the increased presence of autolyzed calpain-2 activity in OW carcasses.

Several studies have reported TBARS value thresholds for off-flavor detection (Tarladgis et al., 1960; Campo et al., 2006) with a commonly used concentration of 1 malondialdehyde/kg meat (McKenna et al., 2005) being used as a benchmark. In the current study, there was an observed increase in TBARS values throughout the display time period, however the values were still relatively low and below the threshold of detectable off-flavors. Similar results were observed by McKenna et al. (2005), who reported a lag phase before the accumulation of oxidative rancidity by-products. Sawyer et al. (2007) also reported an increase in TBARS values through the retail display, and similarly did not see a difference between the deep and superficial locations. Conversely, Puga et al. (2019) reported the deep location of the top round had greater TBARS values on average on d 4 compared to the superficial location, however all values were below the off-flavor threshold. Given low TBARS values, discoloration to top round steaks are likely not related to lipid oxidation.

Color variation within the SM has been reported, with the deep section being lighter in color, redder in appearance, and more yellow (Lee et al., 2007). The current research demonstrates a similar trend that is further amplified by carcass weight with the deep location of the steaks from OW carcasses being the palest of the locations evaluated across all days. The SM muscle often has elevated L* values compared with other muscles, but this trait alone does not fully account for decreased color stability (McKenna et al., 2005). Steaks from OW carcasses in the current study had greater average b* values through the first day of retail display, this is consistent with McKenna et al. (2005). In other work, Sammel et al. (2002b) reported the deep location maintained a more yellow appearance throughout the retail display period. In contrast, Fevold et al. (2019) reported steaks from heavy carcasses in their study were redder in appearance than those from the lightweight carcasses. In the current study, the superficial location of steaks remained redder throughout the retail display period, which is in agreement with previous research looking at the SM muscle (Sammel et al., 2002a; Seyfert et al., 2006). The difference in temperature and pH decline of the superficial and deep locations are contributing factors to the color difference between the two locations. The current study, similar to previously published work (Sammel et al., 2002a; Seyfert et al., 2006; Nair et al., 2016), reported steaks had more desirable color on the initial observations and the favorable appearance diminished throughout the retail display period. Hector et al. (1992) reported a rapid pH decline postmortem to lead to protein denaturation and subsequently lighter colored muscle. Greater discoloration of the deep location is an effect of protein denaturation as a result of the delayed temperature and accelerated pH decline.

In the current study, it was observed that OW carcasses had a greater percentage surface discoloration over time based on subjective color evaluations. The observed difference in objective color scores averaged over the retail display between the deep and superficial locations of L* was 6.0 and a* had a difference of 3.83. Zhu and Brewer (1999) reported that a change in L* of 0.4 and a* of 0.6 were observable in subjective color evaluations, therefore it is likely that consumers would be able to distinguish between the two muscle locations.

Previous literature varies on the impact of carcass weight on shear force values of loin steaks from AW and OW carcasses. For instance, Foster et al. (2019) reported increased carcass weights (431.4–476.3 kg) and ribeye size can have negative impacts on tenderness compared with steaks sourced from AW cattle (340.7–385.6 kg). Moreover, Agbeniga and Webb (2018) reported no difference in tenderness between carcass weights (AW ≤ 260 kg vs. OW ≥ 290 kg), whereas Djimsa et al. (2018) reported steaks fabricated from OW carcasses were more tender. Similar to outcomes of with Fevold et al. (2019) in the current study, no differences were observed in the cook time, overall cook loss, and WBSF values of top round steaks sourced from AW and OW carcasses. In addition, the 2017 National Beef Tenderness Survey reported 65% of top round steaks in a retail setting had values that were considered very tender with WBSF less than 34.1 N (Martinez et al., 2017). Steaks from both the AW and OW treatments were below the threshold of USDA *Certified Very Tender* level of <3.9 kg (ASTM, 2011). These findings differed from Sawyer et al. (2007) and Puga et al. (2019) where there were differences observed by location in the steak for WBSF values.

## CONCLUSION

The current study suggested there are meaningful differences between the temperature and pH decline relationships in the top rounds among carcasses of different weights. Results indicate that OW carcasses present a challenge in terms of a delayed temperature declined combined with an accelerated pH decline in the top round subprimal. Alternative cooling or fabrication options may need to be considered to better accommodate the continuing trend of increasing beef carcass size in order to optimize the quality of the top round subprimal. Further research should be conducted to look at the impact of aging duration on top rounds from AW and OW carcasses for extended periods of time and the subsequent impacts on steak quality. Extending the aging period could amplify color differences of deep and superficial locations of AW and OW carcasses. Finally, research should be conducted to evaluate the difference in eating quality of the deep and superficial location of top round steaks.

## Supplementary Material

txaa199_suppl_Supplementary_Figures
