# Supplementary material for: Impact of beef carcass size on chilling rate, pH decline, display color, and tenderness of top round subprimals
Source: Transl Anim Sci. 2020 Oct 30;4(4):txaa199. doi: 10.1093/tas/txaa199 (PMC7733321; doi:10.1093/tas/txaa199)

**Supplementary Figures.**

Supplementary Fig. 1. Error estimates and confidence intervals of the (A) OS superficial, (B) OS deep, (C) AW superficial, and (D) AW deep temperature decline graphs.


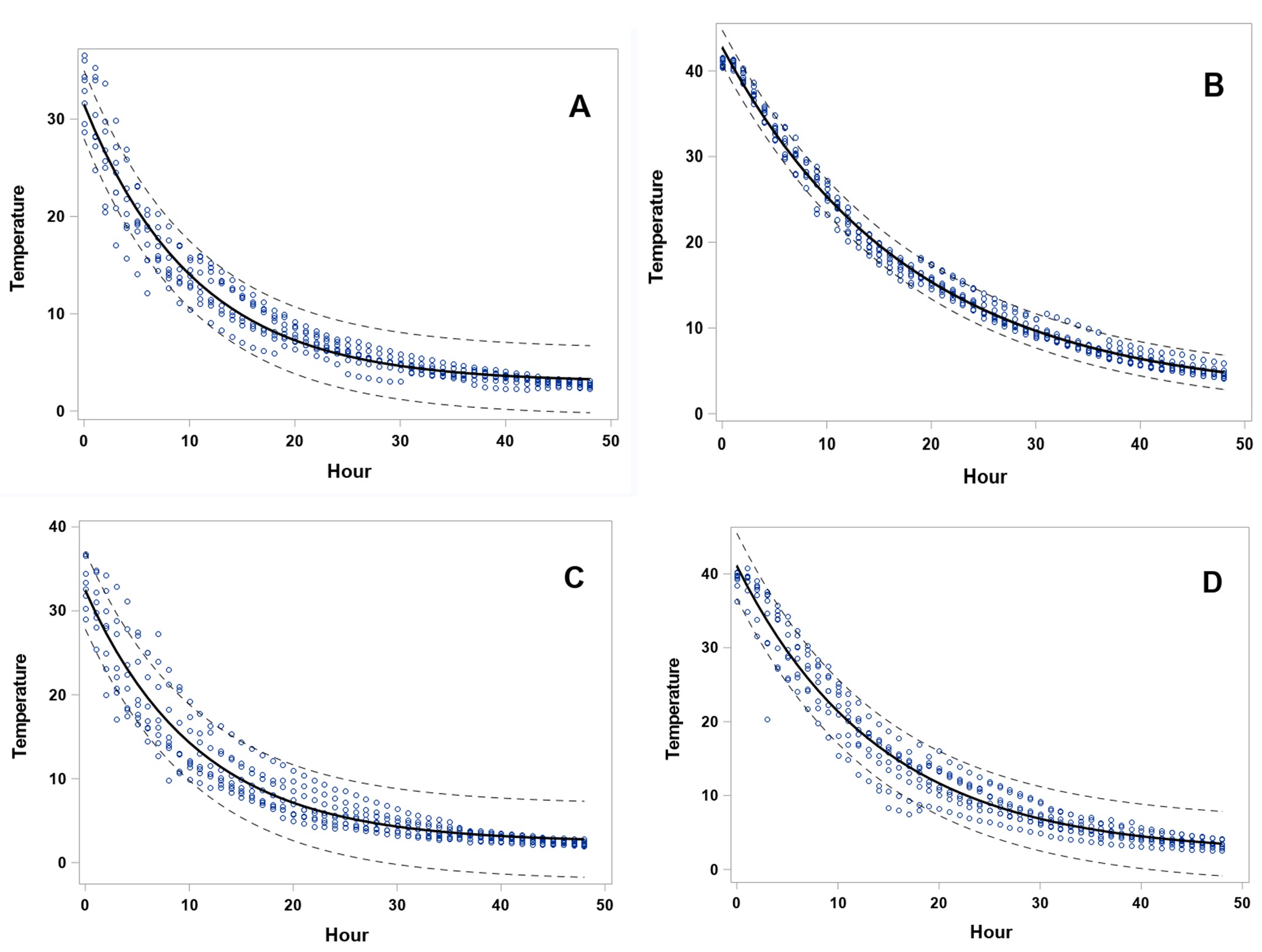


Supplementary Fig. 2. Error estimates and confidence intervals for pH of the (A) OS superficial, (B) OS deep, (C) AW superficial, and (D) AW deep pH decline graphs.


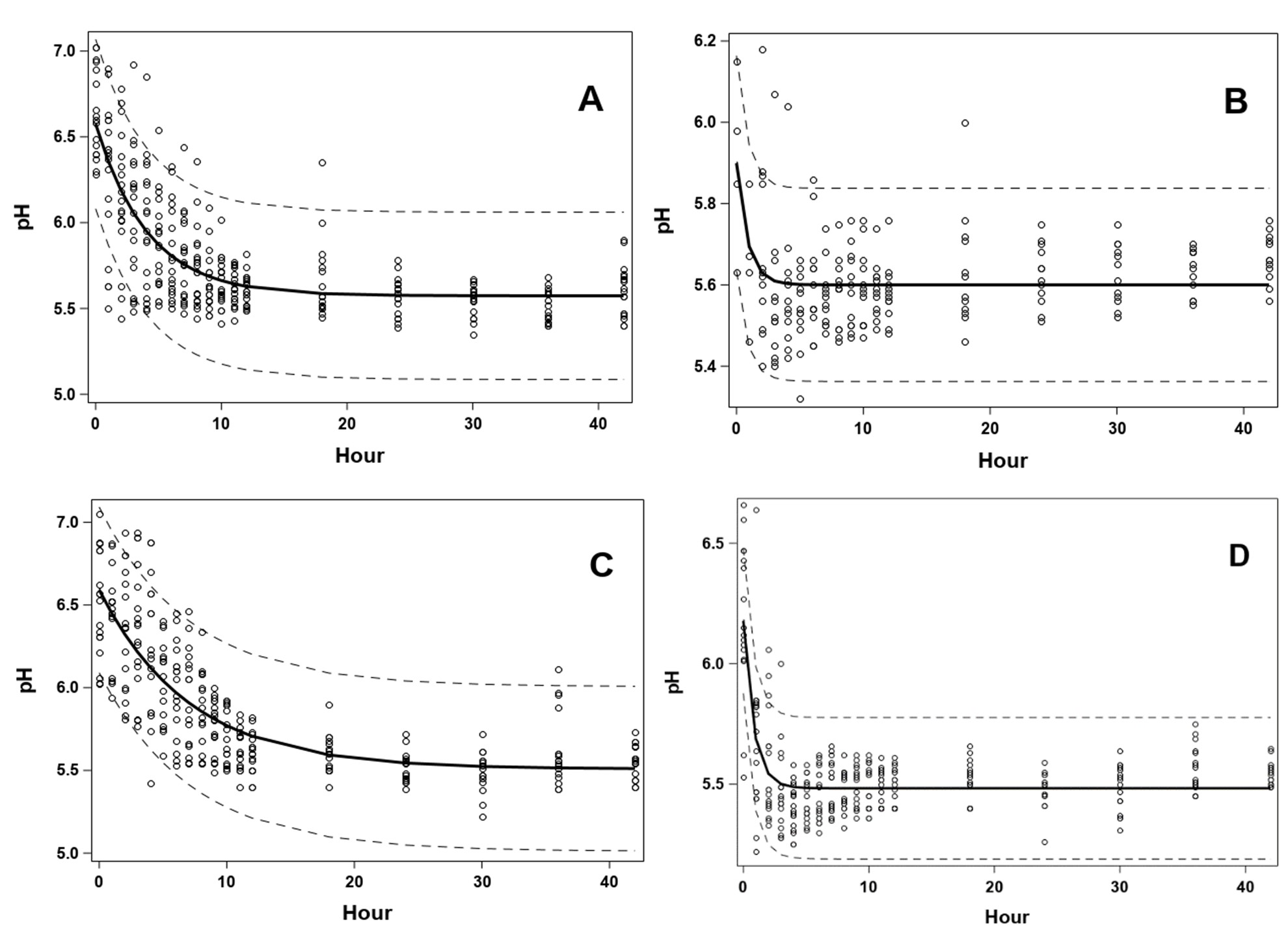

Supplement: txaa199_suppl_Supplementary_Figures [file txaa199_suppl_supplementary_figures.docx]
